# Emerging functions of C/EBPβ in breast cancer

**DOI:** 10.3389/fonc.2023.1111522

**Published:** 2023-01-25

**Authors:** Megan G. Matherne, Emily S. Phillips, Samuel J. Embrey, Caitlin M. Burke, Heather L. Machado

**Affiliations:** ^1^ Department of Biochemistry and Molecular Biology, Tulane School of Medicine, New Orleans, LA, United States; ^2^ Tulane Cancer Center, Louisiana Cancer Research Consortium, New Orleans, LA, United States

**Keywords:** C/EBPβ, breast cancer, transcription factor, immune cells, gene regulation

## Abstract

Breast tumorigenesis relies on complex interactions between tumor cells and their surrounding microenvironment, orchestrated by tightly regulated transcriptional networks. C/EBPβ is a key transcription factor that regulates the proliferation and differentiation of multiple cell types and modulates a variety of biological processes such as tissue homeostasis and the immune response. In addition, C/EBPβ has well-established roles in mammary gland development, is overexpressed in breast cancer, and has tumor-promoting functions. In this review, we discuss context-specific roles of C/EBPβ during breast tumorigenesis, isoform-specific gene regulation, and regulation of the tumor immune response. We present challenges in C/EBPβ biology and discuss the importance of C/EBPβ isoform-specific gene regulation in devising new therapeutic strategies.

## 1 Introduction

Transcription factors regulate gene expression by recognizing and binding specific DNA sequences such as promoters and enhancers, resulting in either direct or indirect activation or repression of gene transcription. Transcriptional regulation of gene expression is a fundamental biological process that is often modified during cancer initiation, progression, and metastasis (1, 2). Cancer cells adopt mechanisms to escape immune surveillance, evade growth signals, and invade surrounding tissues, and transcription factors are instrumental in driving gene expression programs that aid in acquiring these properties. While much is known about the signaling pathways affecting breast cancer cell growth and apoptosis, fewer studies have addressed transcription factor regulation of gene expression in both the tumor cells and the surrounding microenvironment. CCAAT/enhancer binding protein beta (C/EBPβ), which has long been postulated to promote tumorigenesis and metastasis (3, 4), has more recently emerged as an important transcription factor in both tumor and immune cells.

C/EBPβ is a transcription factor that is one of six members of the C/EBP family, each with highly conserved DNA-binding and basic leucine zipper domains, which form homodimers or heterodimers with one another to bind DNA (5, 6). Three different protein isoforms of C/EBPβ may be translated from the intronless gene: liver-enriched activating protein (LAP) 1 (also referred to as *LAP), LAP2 (also referred to as LAP), and liver-enriched inhibitory protein (LIP) (5). LAP1 and LAP2 act as transcriptional activators, but LIP lacks the N-terminal transactivation domain while retaining dimerization and instead acts as a dominant negative transcriptional repressor (3, 5). Post-translational modifications are responsible for dictating the transcriptional activity, subcellular localization, and protein-protein interactions of C/EBPβ. C/EBPβ is naturally held in a repressed state by its two regulatory domains that sterically hinder its transactivation domain (6, 7). Sequential phosphorylation at Thr188 followed by Ser184 or Thr179 results in a conformational change where the intramolecular repression of the transactivation domain is relieved, allowing for transcriptional activation. Phosphorylation of C/EBPβ is regulated by many different pathways (8, 9) including Ras-MAPK (10, 11), protein kinases A and C (12), Ca^2+^/calmodulin dependent protein kinase (13), glycogen synthase kinase 3β (GSK3β) (14), and CDK-cyclin A complexes (15).

C/EBPβ regulates genes involved in proliferation, differentiation, tissue homeostasis, and the immune response in multiple tissue types (4, 8, 16). In the mouse mammary gland, C/EBPβ is required for proper mammary gland development, cellular differentiation, and stem cell activity (17–19). The gene encoding C/EBPβ is generally not mutated in human breast cancers (20). However, increased expression of C/EBPβ is associated with more proliferative and aggressive tumors (21), and a small subset of mammary neoplasms has been shown to have amplification of *Cebpb* (22). Increased C/EBPβ mRNA and protein expression has been associated with triple negative breast cancer (TNBC) (23, 24), although it is unknown whether C/EBPβ expression is altered in other breast cancer subtypes. In syngeneic tumor models, knockdown of C/EBPβ results in decreased tumor latency (25) and tumors with unique histopathologies that include increased inflammation and necrosis (24). The dominant negative isoform LIP is of particular interest, as it has been shown to induce proliferation of mammary epithelial cells (17, 26) and is highly expressed in ER^neg^ tumors with a high proliferative index, compared to ER^+^ breast cancers (4, 26, 27). LIP-deficient transgenic mice (*Cebpb^ΔuORF^*) have decreased spontaneous tumor incidence and delayed tumor onset (28–30), whereas mice with elevated LIP (*Wap-LIP* or *Cebpb^-/L^*) have increased tumor development (4, 31). These studies exemplify the importance of C/EBPβ isoform-specific regulation of gene expression. In this review, we will discuss several mechanisms by which C/EBPβ modulates breast cancer progression, and its implications in therapeutics.

## 2 Growth-regulatory functions

Sustained proliferative signaling and evasion of growth suppression during cell cycle progression are important processes to ensure the success of tumor progression (32). C/EBPβ has been suggested to mediate these processes through interactions with key cell cycle regulators, including cyclin D1 (33), which is required for cell cycle progression through the G1 phase (34). The cyclin D1 gene (*CCND1*) is frequently amplified in human breast cancer, and similar to C/EBPβ, has a critical role in the differentiation of mammary epithelial cells during pregnancy (35–37). While mammary epithelial cells from C/EBPβ^-/-^ mice have a block in cell cycle progression at the G1/S transition, cyclin D1 levels remained unchanged, suggesting that C/EBPβ regulation of cell cycle progression is not dependent on cyclin D1 (38). However, cyclin D1 binds to and activates LAP1 to promote mammary epithelial cell differentiation independent of LAP2 and LIP, suggesting a unique interplay between C/EBPβ and cyclin D1 is required for cell differentiation (39). The block in cell cycle progression was associated with decreased cyclin E expression, increased p27 stability, and decreased CDK2 activity (38). The retinoblastoma protein (Rb), a tumor suppressor and G1 checkpoint regulator, has also been shown to directly activate C/EBPβ during cell cycle progression. In addition, C/EBPβ can bind and activate Rb, causing aberrant function of the protein in a pro-tumorigenic fashion (40–43). Furthermore, C/EBPβ has been shown to bind E2F1 and E2F2 to activate E2F targets through CBP/p300, ultimately recruiting chromatin remodeling complexes (44–46). The effect of C/EBPβ binding to either Rb or E2F is not limited to modulating gene transcription but may have an anti-tumorigenic effect by promoting cell cycle exit. In MCF10A cells, the Rb:E2F-dependent senescence pathway requires C/EBPβ for program activation (43). While a direct interaction with Rb:E2F and C/EBPβ has yet to be shown, C/EBPβ acts synergistically with Rb:E2F to repress S-phase associated genes, and C/EBPβ-null cells fail to enter senescence (43, 47).

Additional seemingly paradoxical functions of C/EBPβ are seen in other growth regulation pathways related to transforming growth factor beta (TGFβ). TGFβ normally functions as a growth inhibitor for epithelial cells, acting as a tumor suppressor. In response to TGFβ, LAP2 complexed with FoxO-Smad activates the p15INK4b promoter, while LAP2 complexed with E2F4/5-Smad to repress c-Myc, providing key anti-growth signals. During the switch to metastatic cancer, tumor cells evade growth inhibitory functions of TGFβ by upregulating LIP, causing a block in C/EBPβ-induced p15INK4b activation and relieving c-Myc repression (48). As mentioned previously, TNBC cells have been shown to express a high LIP : LAP ratio, supporting an oncogenic role for LIP (4, 27, 48). These diverse phenotypes demonstrate both anti-tumor and pro-tumor properties of C/EBPβ, which can be attributed to isoform-specific gene regulation (9, 26, 44).

## 3 Epithelial-mesenchymal transition

In addition to growth-promoting and growth-inhibitory functions, C/EBPβ has been associated with epithelial-mesenchymal transition (EMT), a process where cancer cells acquire mobility and invasive properties due to loss of cell-to-cell junctions (49). TGFβ is a well-known inducer of EMT, and studies have shown that loss of C/EBPβ during the TGFβ response promotes EMT by reducing C/EBPβ-mediated *CDH1* (E-cadherin) transcription (25), suggesting that loss of C/EBPβ is required for EMT. Additionally, in a mouse model used to study the effect of obesity on post-menopausal hormone receptive negative breast cancer, obesity-induced C/EBPβ chromatin binding resulted in elevated expression of MMP9 and claudin-1 (50). In mammary epithelial cells, LIP, but not LAP1/2, is induced by *H-Ras* and was shown to inhibit singleminded 2 (SIM2) gene expression (51). SIM2 is a tumor suppressor that is downregulated in mammary epithelial cells at periods where LIP is the dominant C/EBPβ isoform. SIM2 represses MMP3 and SLUG (52), both of which induce EMT (53, 54). Knockdown of SIM2 is associated with decreased E-cadherin and increased MMP2, N-cadherin and vimentin (55). These reports suggest that LIP may indirectly induce EMT by repressing SIM2. A recent study shows that LIP promotes cell migration in untransformed MCF10A cells and LAP expression reduces migration in TNBC cell lines (BT-20 and BT-549) (56). In contrast, overexpression of LAP2 in MCF10A cells *in vitro* has been shown to induce EMT-like morphologies, accompanied by delocalized E-cadherin and increased vimentin (57). Several studies have shown that LAP2 binds to and activates cyclooxygenase-2 (COX-2) gene expression (58–65), which promotes TGFβ-induced EMT in a PGE_2_-dependent manner (66). Together, these reports suggest C/EBPβ isoform-specific roles in regulating EMT, although further studies are required to define these mechanisms.

## 4 Tumor-promoting inflammation

### 4.1 Tumor-derived C/EBPβ

Tumor-promoting inflammation is another hallmark of cancer, where cancer cells adopt inflammatory mechanisms to promote their growth and survival (32). C/EBPβ, initially identified as nuclear factor for interleukin-6 expression (NF-IL6) (67, 68), has important roles in mediating the inflammatory response. Early studies indicated that C/EBPβ binds to an IL-1 response element in the IL-6 promoter to drive IL-6 and IL-8 transcription. C/EBPβ and other C/EBP family members can directly interact with the Rel homology domain between NF-κB subunits p50, p65 and c-Rel, stabilizing NF-κB, leading to synergistic transcriptional activation of IL-6 and IL-8 (69, 70). C/EBPβ can also positively regulate NF-κB by binding and inactivating IκBα, the canonical inhibitor of NF-κB (71). In breast cancer cells, C/EBPβ can act in synergy with STAT3 to activate c-Jun activation domain-binding protein (Jab)-1, which regulates cycle control, apoptosis, and DNA repair (72). More recently, STAT3 was shown to stabilize C/EBPβ in *H-Ras* transformed mammary epithelial cells, to cooperatively induce the transcription of cancer-promoting inflammatory cytokines (70, 73). C/EBPβ was also shown to directly bind to the STAT5 promoter, facilitating TNBC cell growth and invasion *in vitro* (23). These results demonstrate that with other key transcription factors, C/EBPβ coordinately promotes the induction of various inflammatory cytokines during tumor progression.

Chemokine signaling facilitates leukocyte recruitment and activation and can dictate the balance between the pro- and anti-tumor immune response. In the tumor microenvironment, tumor and immune cells, such as tumor-infiltrating macrophages, secrete chemokines to promote immune evasion, growth and survival of tumor cells, angiogenesis, and metastasis (74). *Cebpb^-/-^* mice have defective helper T cell function and lymphoproliferative diseases (75), which are in part due to alterations in cytokine and chemokine production. Knockdown of C/EBPβ in mouse mammary tumor 4T1 cells results in increased expression of various chemokines such as in CCL6, CCL7, CCL8, CCL12, CCL27, CCL28, and CXCL16 expression, although whether C/EBPβ directly inhibits these chemokines was not addressed (24). Other studies have shown that tumor-derived C/EBPβ directly activates various chemokines, including CCL2, CCL5, CXCL12 and CXCR4 (76–80). In contrast, LIP was shown to bind to and inhibit the CCL2 promoter (78), and LIP can indirectly activate CXCR4 by inhibiting the CXCR4 repressor YYI (80). These opposing roles for LIP in chemokine activation/inhibition may be due to microenvironment- and tumor context-specificity, however, further studies are required to address these differences. C/EBPβ has also been shown to cooperate with ATF to activate RANKL (81). Notably, while a number of studies have shown altered chemokine expression in cancer cells after C/EBPβ knockdown, few studies have validated whether and how LAP/LIP directly bind to chemokine promoters.

In addition to recruiting tumor-promoting immune cells, breast cancer cells evade cytotoxic effector cells by downregulating the expression of receptor-bound recognition proteins. Transformed cells actively downregulate immunogenic surface receptors to avoid immune recognition and destruction by cytotoxic CD8^+^ T cells (82–84). A recent study showed that deletion of C/EBPβ in human breast cancer cells (BT-20) results in the de-repression of genes involved in immune visibility, where MHC I and MHC II gene sets were significantly upregulated as compared to wildtype (56). In another study, C/EBPβ-silenced 4T1 cells were shown to have significantly upregulated expression of MHCIIα, MHCIIβ and HLACIIγ (24). While functional antigen presentation assays were not performed, C/EBPβ-silenced 4T1 tumors displayed a significant increase in CD3^+^ lymphocytes *in vivo* (24). Finally, C/EBPβ was shown to repress type I and type II interferon response genes in a mouse model of *Ras*-induced squamous papilloma (85). These studies suggest that C/EBPβ may aide in immune escape, although further investigation is required to define the specific mechanisms.

### 4.2 Myeloid-derived C/EBPβ

While studies have focused on how tumor-derived C/EBPβ inhibits or promotes tumorigenesis, C/EBPβ is also expressed in immune cells, and in particular has important roles in myeloid cells. C/EBPβ has well-established roles in myelopoiesis (86, 87), and overexpression of LAP1 or deletion of C/EBPβ in THP-1 cells causes decreased monocyte proliferation (88). More recently, C/EBPβ was shown to be required for Ly6C^+^ monocyte differentiation into Ly6C^-^ cells, through a mechanism involving C/EBPβ activation of *Nr4a1* (89). In addition, *Cebpb^-/-^* bone marrow-derived macrophages have impaired phagocytic function (75, 90). Macrophage-derived C/EBPβ also modulates the balance between pro- and anti-inflammatory signals during tissue repair. In the wound healing process, pro-inflammatory cytokines such as IL-6, TNFα and IL-1β induce C/EBPβ activation in macrophages, which in turn activates these cytokines in a feedback loop to modulate inflammation (87, 91). While transcription factor regulation of macrophages has been studied in response to injury, less is known about how macrophage C/EBPβ regulates tumor progression. In a syngeneic mouse model of early-stage breast cancer progression, *Cebpb* was highly expressed in numerous macrophage populations identified by single cell RNA sequencing, as well as in *Ly6c2^+^* monocytes, and *S100a8^+^* neutrophils, the latter of which likely give rise to MDSCs (92). In metastatic melanoma, macrophages with high C/EBPβ expression had significantly higher activation of the IL6-JAK-STAT3 signaling pathway (93). These data suggest that C/EBPβ may have crucial roles in regulating the functions of tumor-infiltrating macrophages, although further investigation is required to validate this idea.

C/EBPβ has been shown to be a key transcription factor regulator of MDSCs. C/EBPβ is required for the differentiation and expansion of MDSCs in the bone marrow of septic mice, by inducing miR-21a, miR-21b and miR-181b in a STAT3-dependent manner (94, 95). In tumor models, genetic ablation of C/EBPβ from MDSCs isolated from tumors impairs T-cell suppressor activity (96). C/EBPβ can induce arginase I expression in response to injury (97, 98), providing one potential mechanism for C/EBPβ-mediated immune-suppressive function of MDSCs during cancer progression. In colon cancer cells, Gao et al. identified a long noncoding RNA, termed lnc-C/EBPβ, which binds to LIP to inhibit the activation of C/EBPβ target genes, such as *Arg1*, *Nos2*, *Nox2 and Cox2*, and thus negatively regulates immune-suppressive functions of MDSCs (99). In TNBC, LAP2 was shown to promote the recruitment of MDSCs by activating G-CSF and GM-CSF through a tumor-specific glycolysis-dependent pathway (100, 101). Together, these studies suggest that both tumor- and myeloid-derived C/EBPβ are important regulators of MDSC immune-suppressive function.

## 5 Discussion

**Figure 1:**
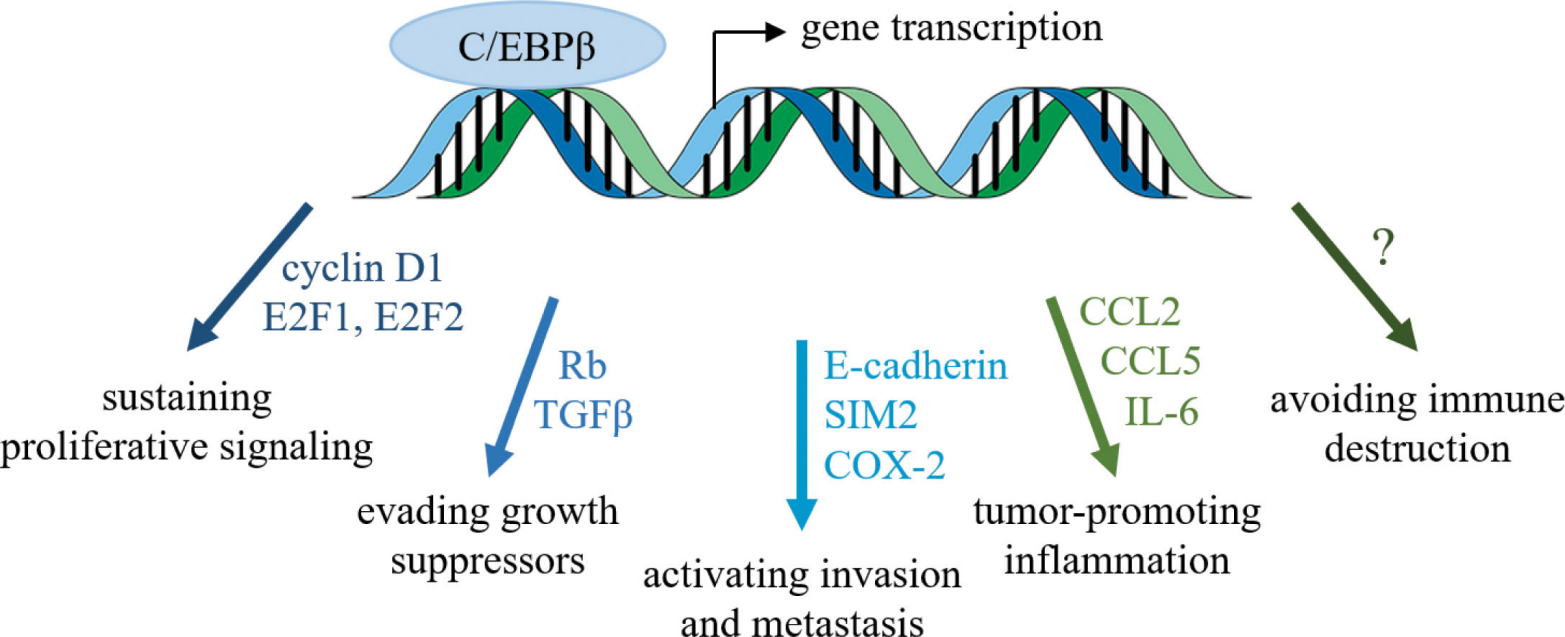
Transcription factor C/EBPβ regulates several hallmarks of cancer. C/EBPβ influences the cell cycle and epithelial-mesenchymal transition of breast cancer cells, as well as the tumor microenvironment.

C/EBPβ has emerged as a critical transcription factor for successful breast tumor progression, promoting cancer cell growth and survival, metastasis, inflammation, and potentially immune evasion (**Figure 1**). C/EBPβ has also been implicated in therapeutic resistance. In colorectal cancer, FOXO1/C/EBPβ/NF-κB signaling is required for CCL20-dependent recruitment of regulatory T cells, which confer chemoresistance to 5-fluorouracil (102). Radiation resistance of nasopharyngeal carcinoma has been attributed to the PGC1α/C/EBPβ/CPT1A axis (103), and C/EBPβ is required for therapeutic resistance in NRF2-activated non-small cell lung cancer (104). Thus, C/EBPβ may be an attractive target in overcoming therapeutic resistance.

While transcription factors have traditionally been considered “undruggable,” emerging studies have focused on overcoming the challenges associated with targeting transcription factors as a therapeutic strategy (105, 106). A recent study demonstrated the ability of a selective peptide C/EBPβ antagonist, ST101, to induce ubiquitin-dependent C/EBPβ degradation, resulting in tumor growth inhibition in xenograft models (107). Similarly, cell-penetrating peptides Bpep and Dpep have been designed to act as leucine zipper decoys with specificity for tumor C/EBPβ (108). Other studies have shown that LIP can be inhibited by pharmacological inhibition of mTORC1, suggesting that rapamycin analogues may be an effective therapeutic strategy (29, 109). Synthetic analogues of helenalin covalently bind and inhibit C/EBPβ and have been shown to reduce proliferation in acute myeloid leukemia cells *in vitro* (110, 111). In non-small cell lung cancer, metformin reduced tumor growth *via* the AMPK/C/EBPβ/PD-L1 axis (112). The efficacy of these small molecule inhibitors in breast cancer has yet to be determined.

Despite emerging studies demonstrating the importance of C/EBPβ in breast cancer progression, much remains to be learned about the gene regulatory networks induced by C/EBPβ during tumor progression. The complexity of C/EBPβ isoform-specific gene regulation (**Table 1**) has largely hindered our understanding of C/EBPβ-induced gene expression in both tumor and immune cells. LAP1/LAP2 and LIP can have opposing functions within the same pathway or program, yet many valuable studies lack details on which isoform of C/EBPβ regulates gene expression and protein function. The lack of isoform-specific antibodies continues to present a significant challenge for both basic science and clinical studies (44). For example, defining isoform specificity at different stages of breast cancer progression may provide insights on whether isoform expression, and downstream targets, are of prognostic value. Despite these limitations, advances in genetic mouse models and gene editing technology has recently allowed for the study of individual isoforms, by using approaches that delete C/EBPβ while simultaneously overexpressing LAP2 or LIP (31). Future studies should focus on understanding isoform-specific functions in both the tumor and immune cells, which will have critical implications for developing therapeutic strategies that target either specific C/EBPβ isoforms, such as LIP, or C/EBPβ-induced target pathways.

**Table 1:** Isoform-specific regulation of gene expression and protein function.

Isoform	Gene or protein	Result	References
LAP1	Binds cyclin D1	Mammary epithelial cell differentiation	(33, 38, 39)
LAP2	Binds FoxO-Smad complex	Tumor suppressor p15INK4b promoter is activated	(48)
	Binds E2F4/5-Smad complex	Proto-oncogene c-Myc promoter is repressed	(48)
	Delocalized expression of E-cadherin and increased expression of vimentin	EMT phenotype is expressed	(57)
	Increased expression of E-cadherin and decreased expression of vimentin	Epithelial phenotype is maintained	(25)
	Activates COX-2 expression	EMT phenotype is expressed	(58–66)
LIP	Inhibits LAP2 from binding FoxO-Smad complex	Blocks transcription of tumor suppressor p15INK4b	(48)
	Inhibits LAP2 from binding E2F4/5-Smad complex	Proto-oncogene c-Myc is expressed	(48)
	Inhibits SIM2	EMT phenotype is expressed	(51)
	Inhibits CCL2 expression	Reduces pro-inflammatory phenotype	(78, 113)
	Binds and inhibits YY1	CXCR4 expression is activated	(80)
Isoform unknown or not specified	Binds Rb	Activates C/EBPβ, activates Rb	(40, 42)
	Binds E2F1 and E2F2	Recruits chromatin remodeling complexes	(45, 46)
	Interacts with Rb:E2F	Induces cell senescence	(43, 47)
	Regulates Rb/E2F/cyclin E pathway	Reduces monocyte proliferation	(88)
	Binds NF-κB	IL-6 and IL-8 expression is activated	(67–69, 76, 100, 114, 115)
	Binds IκBα	NF-κB is not inhibited	(71)
	Interacts with STAT3	Stabilizes C/EBPβ, activates Jab-1	(70, 72, 73)
	Activates STAT5 expression	JAK/STAT pathway is activated	(23)
	Activates CCL2 and CCL5 expression	Promotes metastasis	(76–78, 116–119)
	Regulates CXCL12 expression	CXCR4/CXCL12 axis promotes metastasis	(79, 80)
	Inhibits MHCI and MHCII	Reduced antigen recognition	(24, 56)
	Activates arginase I expression	Macrophages take on anti-inflammatory phenotype	(97, 98)

## Author contributions

MM: Manuscript writing, conception of the topic. EP: Manuscript writing. SE: Manuscript writing. CB: Manuscript editing and revision. HM: Conception of the topic, manuscript editing and revision, funding. All authors contributed to the article and approved the submitted version.
